# The professionalism disconnect: do entering residents identify yet participate in unprofessional behaviors?

**DOI:** 10.1186/1472-6920-14-60

**Published:** 2014-03-27

**Authors:** Alisa Nagler, Kathryn Andolsek, Mariah Rudd, Richard Sloane, David Musick, Lorraine Basnight

**Affiliations:** 1 Duke University Hospital, Center for the Study of Aging and Human Development, Durham, NC, USA; 2 Office of Medical Education, Vidant Medical Center/East Carolina University, Greenville, USA; 3 Duke University Hospital – Graduate Medical Education, PO Box 3951, DUMC, Durham, NC, USA

## Abstract

**Background:**

Professionalism has been an important tenet of medical education, yet defining it is a challenge. Perceptions of professional behavior may vary by individual, medical specialty, demographic group and institution. Understanding these differences should help institutions better clarify professionalism expectations and provide standards with which to evaluate resident behavior.

**Methods:**

Duke University Hospital and Vidant Medical Center/East Carolina University surveyed entering PGY1 residents. Residents were queried on two issues: their perception of the professionalism of 46 specific behaviors related to training and patient care; and their own participation in those specified behaviors. The study reports data analyses for gender and institution based upon survey results in 2009 and 2010. The study received approval by the Institutional Review Boards of both institutions.

**Results:**

76% (375) of 495 PGY1 residents surveyed in 2009 and 2010 responded. A majority of responders rated all 46 specified behaviors as unprofessional, and a majority had either observed or participated in each behavior. For all 46 behaviors, a greater percentage of women rated the behaviors as unprofessional. Men were more likely than women to have participated in behaviors. There were several significant differences in both the perceptions of specified behaviors and in self-reported observation of and/or involvement in those behaviors between institutions.

Respondents indicated the most important professionalism issues relevant to medical practice include: respect for colleagues/patients, relationships with pharmaceutical companies, balancing home/work life, and admitting mistakes. They reported that professionalism can best be assessed by peers, patients, observation of non-medical work and timeliness/detail of paperwork.

**Conclusion:**

Defining professionalism in measurable terms is a challenge yet critical in order for it to be taught and assessed. Recognition of the differences by gender and institution should allow for tailored teaching and assessment of professionalism so that it is most meaningful. A shared understanding of what constitutes professional behavior is an important first step.

## Background

Professionalism has been an important tenet of medical education at least since the time of Hippocrates [1]. In the last two decades the Liaison Committee on Medical Education [2], the Accreditation Council for Graduate Medical Education (ACGME) [3], and the American Board of Medical Specialties [4] have required formal training and assessment in professionalism for physicians in training and practice.

Physician professionalism increasingly has been linked to improved patient outcomes [5]. Professionalism lapses lead to more state licensing board actions than a lack of medical knowledge [6,7]. Unprofessional behavior in medical school predicts poor performance in residency [8] and later adverse actions by a licensing board [9-11]. Unprofessional behaviors have also been associated with patient complaints and litigation [12].

Conversely, residents with higher scores on professionalism demonstrate higher in -service training examination and mini-CEX (clinical evaluation exercise) scores, are more likely to complete administrative tasks and are less likely to receive official “warnings” or “probationary status” during their formal training [13].

Defining professionalism in precise terms has not been easy [14]. At present, there are over 200 curricular resources on this topic on the MedEdPortal database offered by the Association of American Medical Colleges. It is evident that many groups have attempted to understand and define professionalism issues related to patient care. This has resulted in a diverse pool of professionalism definitions and standards [14-16]. Most definitions emphasize three general components: the integrity of the individual physician; the ability to conduct appropriate relationships with patients within the context of patient care; and the obligations of physicians to address larger social issues within which care delivery happens and is impacted. Given the emphasis on this topic over the past decade and the abundant available resources that address it, it seems intuitive that learners bring with them subjective perceptions of what constitutes professional behavior. For physicians in training, it seems apparent that definitions of professionalism may vary by individual, demographic, and/or generation. And, the definitions and interpretations of “good professional behavior” offered by medical students and resident physicians in training may also differ from those of their teachers and institutions.

Can professionalism be taught during physician training? The evidence is mixed. Didactic presentations alone are recognized as insufficient to adequately teach professionalism [17]. Professional values are less likely to be taught in formal settings compared with informal ones, when attending physicians are present and provide role modeling of professional behaviors [18]. The powerful influence of the “hidden curriculum” may be more impactful than formal teaching [19]. And, regarding assessment, there are gaps between how students self-assess their professional behaviors and how that behavior is assessed by their supervising faculty [20]. A confounding factor may be the potential disconnect between the perceived unprofessinalism of behaviors and participation in those same behaviors.

For the purposes of this project, the authors were interested in the commonly encountered, day-to-day professionalism issues within clinical practice and the influences on physician training which have been frequently characterized as the “hidden” or “informal” curriculum [21,22]. Cohen suggests professionalism “is a way of acting” [23] or what Williams characterizes as the “humdrum day- in, day- out, everyday work that is the real satisfaction of the practice of medicine [24]”. These issues change with time. Currently, phenomona such as the emergence of social media, duty hour restrictions and electronic medical records pose new challenges to healthcare and definitions and adherence to professionalism. How to identify and understand the professionalism challenges faced by physicians in training should help training institutions set clear expectations regarding professional conduct and provide standards to evaluate resident behavior.

The goal of this study was to identify entering first year resident physicians’ (PGY1) perspectives on professionalism issues and their own behaviors that may contradict these perceptions. The findings are intended to inform professionalism teaching and assessment practices and to identify contemporary issues which may impact learners. In addition, we explored how the professionalism items varied by PGY1, gender and by institution. Brody School of Medicine at East Carolina University and the Vidant Medical Center are part of a regional health system (Vidant Health) which provides tertiary care, yet emphasizes primary care, comprehensive medical education and community-based health services to a 29-county region in eastern North Carolina. Duke University Hospital is a 924-bed academic tertiary and quaternary care facility located in Durham, North Carolina. Since its establishment in 1930, the hospital has grown from a small regional hospital to an academic medical center.

## Methods

Two sponsoring institutions, Duke University Hospital (DUH) and Vidant Medical Center/East Carolina University (VMC/ECU) surveyed entering first year residents (PGY1s) at the start of their Graduate Medical Education training in 2009 and 2010. At the time of the study, DUH had over 950 residents training in one of 77 ACGME programs, and 60 internally sponsored programs. VMC/ECU had 340 residents training in one of 28 ACGME programs, and 5 internally sponsored programs. We administered a survey which queried PGY1 residents on their perceptions of the professionalism of 46 specific behaviors related to training and patient care, and whether they had observed and/or participated in those behaviors. Demographic data were also collected. An existing survey which had been utilized in a similar fashion and meaningful results published in the medical education literature was modified with permission of the authors [25]. The survey included behaviors addressing professionalism (some of which have been addressed in other studies) to capture what the PIs, through literature and observation, identified as timely and critical to physician training [26]. Behaviors ranged from egregious (making fun of patients) to controversial (attending a dinner sponsored by a pharmaceutical company). The study was found “exempt” by the Institutional Review Boards of Duke University School of Medicine and East Carolina University.

The survey was administered either electronically via commercially available electronic survey software, or via paper hard copy. All data were entered into the computerized survey program for eventual analyses. Residents were told that participation was voluntary and were asked to create a unique identifier for the purposes of matching participant responses over the entire course of their residency training. Participants were asked to rate their perception of the professionalism of each of the 46 scenarios using five level ordinal response categories (1 = unprofessional; 2 = somewhat unprofessional; 3 = neutral; 4 = somewhat professional; 5 = professional). For the data analyses, survey responses of “professional” and “somewhat professional” were combined into a single category, as were responses of “unprofessional” and “somewhat unprofessional” and responses for “neutral” were not included. In reviewing the distribution of responses, in most cases there were very few in the extreme anchors. In addition there was concern whether responders could distinguish at a meaningful level between “unprofessional” and “somewhat unprofessional” and “somewhat professional” and “professional.” Thus the PIs determined the anchors on either end and the “somewhat” responses were reflecting the same magnitude of ranking. Given these two points, the PIs decided to combine results from “1” with “2” and “4” with “5,” resulting in a 3 level ordinal response metric, “Unprofessional”, “Neutral”, and “Professional”.

Initial review of the data included analysis of the behaviors in which greater than 10% of responders reported having participated. For these behaviors, the PIs were interested in how responders rated these same items. Thus a comparison analysis was done to identify any disconnect between participants’ professionalism rating of these behaviors and their related involvement.

The analysis was structured to describe the distribution of the trends in the 3-level ordinal professionalism response items by the 2 class variables under consideration – gender (male vs female) and institution (DUH vs VMC/ECU). Analysis by PGY level was not relevant for this part of the study. These were evaluated by using the Cochran-Mantel-Haenszel test statistic. The significance level was set at p-value of .05. At the risk of uncovering spurious associations, no adjustment or correction was made for multiple testing because the spirit of this analysis was to discover which professionalism items were associated with gender and institution, and there was no a priori hypotheses entering the analysis. A Cronbach Alpha was run to measure internal consistency.

Two open-ended, qualitative questions were included in the survey:

1. What is the most important issue of professionalism you believe you and other residents face in your specialty?

2. How do you think professionalism can best be assessed?

Grounded theory methods were used to analyze the qualitative data. Data were extracted from the open-ended questions using open coding to identify recurring themes. There were multiple cycles of reading the comments, using the constant comparative method to group concepts into themes. Once continued review of the data revealed no unrecognized themes the reviewers agreed saturation had been reached [27].

Two authors (AN and MR) independently coded all comments. Coding discrepancies were discussed and themes revised to better represent the data. Open-ended comments were not compared across institutions or gender because initial analysis indicated no major differences; instead these data were treated as a single set.

## Results

The survey was administered to 495 entering residents over the two years of the study. A total of 375 residents responded, for an overall response rate of 76%. Of the respondents, 155 (41%) were women and 220 (59%) were men. By institution, the response rates were similar, with VMC/ECU (153, 85%) having a somewhat higher overall response rate than DUH (222, 73%). However, more residents participated from DUH, as the larger institution. The overall response rate across the board at both institutions was somewhat higher for year two (78% DUH, 94% VMC/ECU) than year one. (68% DUH, 75% VMC/ECU). See Table 1.

**Table 1:** Response rate

	**Duke**	**VMC/ECU**	**Total**
**2009**	93/137 (68%)	65/87 (75%)	158/224 (71%)
**2010**	129/166 (78%)	88/94 (94%)	217/260 (83%)
**Total**	222/303 (73%)	153/181 (85%)	375/484 (77%)

Responses to each of the 46 survey items are displayed in Table 2.

**Table 2:** Aggregate survey results

**Behavior**	**Professionalism rating**			**Observed or participated**		
	**Un/Somewhat professional**	**Neutral**	**Some/Fully professional**	**Neither observed or participated**	**Observed**	**Participated**
**Attended a "drugrep" (pharma-sponsored) dinner or social event**	165 (44%)	153 (40.8%)	53 (14.1%0	79 (21.1%)	90 (24.0%)	212 (56.5%)
**As a woman, wore clothing to the hospital which revealed exposed midriff, cleavage or thighs**	347 (92.5%)	18 (4.8%)	6 (1.6%0	143 (38.1%)	233 (62.1%)	5 (1.3%)
**Wore wrinkled shirts or pants, tennis shoes, cargo pants to the hospital**	325 (86.7%)	39 (10.4%)	7 (1.9%)	101 (26.9%)	191 (50.9%)	89 (23.7%)
**Wore a white coat which was in poor condition (e.g. wrinkles, stains, tears in pockets)**	301 (80.3%)	64 (17.1%)	5 (1.3%)	84 (22.4%)	152 (40.5%)	145 (38.7%)
**"Blocked" an admissions you thought was inappropriate**	195 (52%)	111 (29.6%)	63 (16.8%)	150 (40.0%)	172 (45.9%)	59 (15.7%)
**Celebrated a "blocked" admission**	322 (85.9%)	43 (11.5%)	5 (1.3%)	159 (42.4%)	181 (48.3%)	41 (10.9%)
**Disparaged the ER team/outpatient doctor to others for missed findings later discovered on the floor**	348 (92.8%)	16 (4.3%)	5 (1.3%)	152 (40.5%)	197 (52.5%)	32 (8.5%)
**Friended a patient on Facebook**	338 (90.1%)	30 (8%)	3 (0.8%)	306 (81.6%)	70 (18.7%)	5 (1.3%)
**Dated a supervising attending**	326 (86.9%)	41 (10.9%)	4 (1.1%)	274 (73.1%)	103 (27.5%)	4 (1.1%)
**Dated an attending on another service**	204 (54.4%)	146 (38.9%)	21 (5.6%)	256 (68.3%)	119 (31.7%)	6 (1.6%)
**Had coffee, lunch or a drink with a patient**	251 (66.9%)	104 (27.7%)	15 (4%)	295 (78.7%)	72 (19.2%)	14 (3.7%)
**Dated a patient**	359 (95.7%)	8 (2.1%)	4 (1.1%)	320 (85.3%)	56 (14.9%)	5 (1.3%)
**Discussed patient information in a hospital public space (e.g. elevator, cafeteria, parking lot, etc.)**	351 (93.6%)	16 (4.3%)	4 (1.1%)	123 (32.8%)	167 (44.5%)	91 (24.3%)
**Had a nonmedical/personal conversation in a patient corridor (e.g. discussing evening plans)**	185 (49.3%)	146 (38.9%)	39 (10.4%)	100 (26.7%)	96 (25.6%)	185 (49.3%)
**Treated staff, technicians, coordinators, etc. differently than physicians**	326 (86.9%)	37 (9.9%)	8 (2.1%)	156 (41.6%)	202 (53.9%)	23 (6.1%)
**Made fun of a patient to a colleague (e.g. made a derogatory comment about a patient while they were under anesthesia)**	359 (95.7%)	8 (2.1%)	4 (1.1%)	166 (44.3%)	188 (50.1%)	27 (7.2%)
**Made a disparaging comment about a patient on Facebook, blog, etc.**	360 (96%)	6 (1.6%)	4 (1.1%)	292 (77.9%)	86 (22.9%)	3 (0.8%)
**Made a disparaging comment about a student, resident, attending, other member of the healthcare team on Facebook, blog, etc.**	361 (96.3%)	6 (1.6%)	4 (1.1%)	281 (74.9%)	94 (25.1%)	6 (1.6%)
**Skipped a lecture or talk in which attendance was required and no truly urgent patient care issue needed attention**	332 (88.5%)	32 (8.5%)	7 (1.9%)	159 (42.4%)	147 (39.2%)	75 (20.0%)
**Arrived late to rounds for nonclinical reasons**	342 (91.2%)	25 (6.7%)	4 (1.1%)	147 (39.2%)	157 (41.9%)	77 (20.5%)
**Shared an answer with a peer during an examination**	358 (95.5%)	9 (2.4%)	4 (1.1%)	295 (78.7%)	81 (21.6%)	5 (1.3%)
**Cheated on an exam because there wasn't time to study**	362 (96.5%)	5 (1.3%)	3 (0.8%)	305 (81.3%)	73 (19.5%)	3 (0.8%)
**Used material from the web (ppt slides, papers, etc.) to pass off as original work (without referencing the author)**	361 (96.3%)	7 (1.9%)	3 (0.8%)	293 (78.1%)	82 (21.9%)	6 (1.6%)
**Encouraged a student to state that they were doctor to expedite patient care**	353 (94.1%)	13 (3.5%)	5 (1.3%)	245 (65.3%)	126 (33.6%)	10 (2.7%)
**Asked a student to discuss with a patient medical or surgical information which was perceived to be beyond their level of knowledge**	354 (94.4%)	12 (3.2%)	3 (0.8%)	263 (70.1%)	104 (27.7%)	14 (3.7%)
**Asked a student to perform a medical or surgical procedure on a patient which was perceived to be beyond their level of skill**	355 (94.7%)	9 (2.4%)	4 (1.1%)	282 (75.2%)	91 (24.3%)	8 (2.1%)
**Performed medical or surgical procedures on a patient beyond perceived level of skill**	354 (94.4%)	13 (3.5%)	3 (0.8%)	283 (75.5%)	73 (19.5%)	25 (6.7%)
**Misrepresented an ordered test as "urgent" in order to get it expedited**	322 (85.9%)	40 (10.7%)	6 (1.6%)	219 (58.4%)	118 (31.5%)	44 (11.7%)
**Reported patient information (labs, test results, exam results) as normal when uncertain of the true results**	359 (95.7%)	7 (1.9%)	3 (0.8%)	260 (69.3%)	104 (27.7%)	17 (4.5%)
**Indicated a test or examination had been completed when it had not (although was intended to be)**	362 (96.5%)	6 (1.6%)	2 (0.5%)	281 (74.9%)	93 (24.8%)	7 (1.9%)
**Falsified patient records (e.g. backdating a note, documenting physical findings not personally obtained, etc.)**	362 (96.5%)	6 (1.6%)	2 (0.5%)	298 (79.5%)	79 (21.2%)	4 (1.1%)
**Avoided caring for a patient because of their race, religion, ethnicity, sexuality, etc.**	360 (96%)	7 (1.8%)	3 (0.8%)	325 (86.7%)	53 (14.1%)	3 (0.8%)
**Signed out patients over the phone**	217 (57.9%)	122 (32.5%)	30 (8%)	177 (47.2%)	121 (32.3%)	83 (22.1%)
**Posted patient information such as "an interesting rash" or other physical finding on the web, Facebook, blog, etc. without permission)**	349 (93.1%)	18 (4.8%)	3 (0.8%)	304 (81.1%)	74 (19.7%)	3 (0.8%)
**Filmed or photographed a patient without their consent**	356 (94.9%)	10 (2.7%)	3 (0.8%)	293 (78.1%)	80 (21.3%)	8 (2.1%)
**Signed out a procedure or task that could have been completed in order to go home as early in the day as possible**	348 (92.8%)	18 (4.8%)	2 (0.5%)	244 (65.1%)	131 (34.9%)	6 (1.6%)
**Logged false duty hours to protect the GME program and or program director**	342 (91.2%)	20 (5.3%)	8 (2.1%)	231 (61.6%)	128 (34.1%)	22 (5.9%)
**Stayed past required shift limits to complete a patient care task which could have been signed out**	174 (46.4%)	140 (37.3%)	56 (14.9%)	187 (49.9%)	91 (24.3%)	103 (27.5%)
**Did not alert one's attending/supervisor that you may have made an error**	352 (93.9%)	12 (3.2%)	3 (0.8%)	289 (77.1%)	80 (21.3%)	12 (3.2%)
**Did not alert one's attending/supervisor that one of your colleagues had made an error that you were aware of**	332 (88.5%)	35 (9.3%)	3 (0.8%)	295 (78.7%)	74 (19.7%)	12 (3.2%)
**Used a drug from the sample drug cabinet for an indigent patient**	148 (39.5%)	122 (32.5%)	97 (25.9%)	207 (55.2%)	113 (30.1%)	61 (16.3%)
**Used a drug from the sample drug cabinet for a friend or family member**	200 (53.3%)	60 (16%)	10 (2.7%)	272 (72.5%)	99 (26.4%)	10 (2.7%)
**Written or called in a prescription for self**	315 (84%)	45 (12%)	10 (2.7%)	289 (77.1%)	71 (18.9%)	21 (5.6%)
**Written or called in a prescription for a friend, colleague, etc. (without seeing the patient or making a note in a patient chart)**	318 (84.8%)	43 (11.5%)	9 (2.4%)	234 (62.4%)	108 (28.8%)	39 (10.4%)
**Accepted a gift from a patient worth < $25**	167 (44.5%)	146 (38.9%)	57 (15.2%)	222 (59.2%)	107 (28.5%)	52 (13.9%)
**Accepted a gift from a patient worth > $25**	296 (78.9%)	66 (17.6%)	6 (1.6%)	286 (76.3%)	89 (23.7%)	6 (1.6%)

Internal consistency as measured by the Cronbach Coefficient Alpha for the 46 items was high at 0.96.

As can be seen in Table 2, a majority of responders rated all 46 specified behaviors as “unprofessional” or “somewhat unprofessional.”

**Figure 1:**
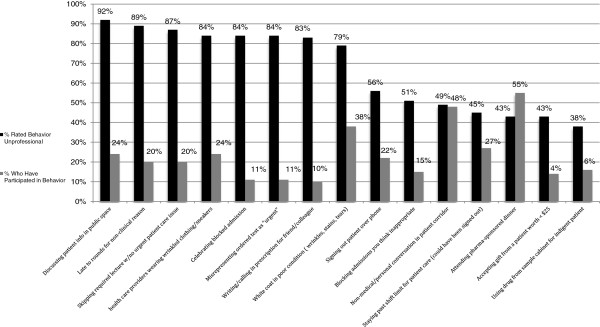
Behaviors reported having participated in by 10% or greater of respondents.

We were especially interested in knowing in which behaviors respondents had personally participated, and yet also felt to be unprofessional. We identified the 15 behaviors with highest levels of reported personal participation. For these 15 behaviors, the data indicate that 10% to 55% reported having participated personally in those behaviors, yet 38% to 92% rated them as unprofessional. These data are displayed in Figure 1.

For these same 15 behaviors, we analyzed the number of individual responders who themselves reported both participating in the behavior and rating it as “unprofessional” or “somewhat unprofessional.” See Table 3. Professionalism around appearance, such as wearing a white coat in poor condition or wrinkled clothes to work, were behaviors a large number of participants rated as “unprofessional” yet had participated in.

**Table 3:** Number of respondents (and percent of total) who reported personal participation and rated as unprofessional

**Behavior**	**Reported personal participation AND rated as unprofessional**	
	**n**	**% of total rating “unprofessional”**
Wore wrinkled shirts or pants, tennis shoes, cargo pants to the hospital	67	20.5
Attended a "drug rep" (pharma-sponsored) dinner or social event	69	41.6
Wore a white coat which was in poor condition (e.g. wrinkles, stains, tears in pockets)	104	34.2
"Blocked" an admissions you thought was inappropriate	12	6.1
Celebrated a "blocked" admission	29	8.9
Discussed patient information in a hospital public space (e.g. elevator, cafeteria, parking lot, etc.)	83	23.4
Had a nonmedical/personal conversation in a patient corridor (e.g. discussing evening plans)	69	36.7
Skipped a lecture or talk in which attendance was required and no truly urgent patient care issue needed attention	61	18.2
Arrived late to rounds for nonclinical reasons	69	20.0
Misrepresented an ordered test as "urgent" in order to get it expedited	29	9.0
Signed out patients over the phone	21	9.6
Stayed past required shift limits to complete a patient care task which could have been signed out	23	13.1
Used a drug from the sample drug cabinet for an indigent patient	2	1.4
Written or called in a prescription for a friend, colleague, etc. (without seeing the patient or making a note in a patient chart)	18	5.6
Accepted a gift from a patient worth < $25	17	10.2

We were also interested in discovering potential gender differences. A greater percentage of women rated the behaviors as unprofessional for all 46 items. These rating differences were statistically significant at the p = .05 level by gender for 14 behaviors. See Table 4.

**Table 4:** Gender differences: professionalism rating

**Q11 Behaviors**	**Professionalism rating**						
	**Unprofessional (1-2)**		**Neutral (3)**		**Professional (4-5)**		**p-value**
	**Female**	**Male**	**Female**	**Male**	**Female**	**Male**	
Women health care providers wearing clothing which reveals exposed midriff, cleavage or thighs	97.37%	90.87%	1.32%	7.31%	1.32%	1.83%	.0423
Men/women health care providers wearing wrinkled shirts or pants, tennis shoes, cargo pants	91.45%	84.47%	7.89%	12.79%	0.66%	2.74%	.0334
Blocking" admissions you think are inappropriate	62.67%	46.79%	26.67%	31.65%	10.67%	21.56%	.0009
Celebrating a "blocked" admission	91.39%	84.40%	7.95%	13.76%	0.66%	1.83%	.0456
“Friending” a patient on Facebook	96.05%	88.13%	3.29%	10.96%	0.66%	0.91%	.0152
Having non-medical/personal conversation in a patient corridor (e.g. discussing evening plans)	56.58%	45.41%	34.87%	42.66%	8.55%	11.93%	.0405
Treating staff, technicians, coordinators, etc. differently than physicians	93.42%	84.02%	5.92%	12.79%	0.66%	3.20%	.0055
Encouraging a student to state that they are a doctor to expedite patient care	98.01%	93.12%	1.32%	5.05%	0.66%	1.83%	.0504
Staying past shift limits to complete a patient care task which could have been signed out	53.64%	42.20%	34.44%	40.37%	11.92%	17.43%	.0266
Writing or calling in a prescription for self	90.07%	81.65%	7.95%	15.14%	1.99%	3.21%	.0417
Accepting a gift from a patient worth > $25	86.75%	75.46%	13.25%	21.76%	0.00%	2.78%	.0032
Dating an attending on another service	65.13%	47.49%	31.58%	45.21%	3.29%	7.31%	.0007
Dating a supervising attending	95.39%	82.65%	3.95%	15.98%	0.66%	1.37%	.0006
Having coffee, lunch or a drink with a patient	79.61%	60.55%	19.08%	33.49%	1.32%	5.96%	< .0001

Regarding participation in these behaviors, men were more likely than women to have participated in 37 of the 46 behaviors; and for two of these behaviors, the differences were statistically significant. Men were significantly more likely than women to report having participated in "making disparaging comments about other members of the healthcare team" (p = .05) and "not alerting a supervisor that a mistake was made" (p = .01) (Table 5).

**Table 5:** Gender differences: observation and participation

**Q12 Behaviors**	**Neither observed or participated**		**Observed**		**Participated**		**p-value**
	**Female**	**Male**	**Female**	**Male**	**Female**	**Male**	
Making disparaging comments about students, residents, attending, other members of the healthcare team on Facebook, blog, etc.)	68.59%	77.78%	30.13%	20.44%	1.28%	1.78%	.0520
Not alerting ones attending/supervisor that you may have made an error	82.05%	71.56%	17.31%	23.56%	0.64%	4.89%	.0126

Finally, we were interested in whether there were differences in respondents’ views based on the training institution they were entering. Regarding whether specific behaviors were perceived as professional, there were statistically significant differences between institutions for 5 of the 46 behaviors and these are displayed in Table 6.

**Table 6:** Institution differences: professionalism rating

**Q11 Behaviors**	**Professionalism rating**						
	**Unprofessional (1-2)**		**Neutral (3)**		**Professional (4-5)**		**p-value**
	**Duke**	**VMC/ECU**	**Duke**	**VMC/ECU**	**Duke**	**VMC/ECU**	
Reporting patient information (labs, test results) as normal when uncertain of the true results	96.11%	100.00%	2.72%	0.00%	1.17%	0.00%	.0447
Not alerting ones attending/supervisor that you may have made an error	94.55%	99.11%	4.28%	0.89%	1.17%	0.00%	.0438
Not alerting ones supervisor that one of your colleagues has made an error and you are aware	87.60%	94.74%	11.24%	5.26%	1.16%	0.00%	.0291
Use drug from sample cabinet for indigent patient	43.92%	31.58%	33.73%	32.46%	22.35%	35.96%	.0043
Attending a "drug-rep" sponsored dinner	58.14%	13.68%	37.60%	49.57%	4.26%	36.75%	< .001

For three of these behaviors, a greater percentage of participants entering VMC/ECU programs rated the behaviors as “unprofessional” or “somewhat unprofessional”; whereas for two behaviors, a greater percentage of participants entering DUH programs rated the behaviors as “unprofessional” or “somewhat unprofessional”. Although not statistically significantly different, there were a greater percentage of VMC/ECU participants than DUH rating the behaviors as unprofessional or somewhat unprofessional for 27 of the 41 remaining behaviors. The greatest difference between the two institutions on any single item was for the behavior “attending a drug-rep sponsored dinner” (rated as professional or somewhat professional; DUH 4.26% and VMC/ECU 36.75%).

There were statistically significant differences between institutions for 11 of the 46 behaviors participants reported they had participated in or observed and these are displayed in Table 7.

**Table 7:** Institutional differences: observation and participation

**Q12 Behaviors**	**Neither observed or participated**		**Observed**		**Participated**		**p-value**
	**Duke**	**VMC/ECU**	**Duke**	**VMC/ECU**	**Duke**	**VMC/ECU**	
Attended a drug-rep sponsored dinner	23.40%	16.53%	26.79%	16.53%	49.81%	66.94%	.0037
Use drug from sample cabinet for indigent patient	58.49%	47.11%	29.43%	28.93%	12.08%	23.97%	.0086
"Blocked" admissions you thought inappropriate	34.34%	52.89%	48.68%	35.54%	16.98%	11.57%	.0011
Celebrated a "blocked" admission	36.60%	55.37%	51.32%	37.19%	12.08%	7.44%	.0007
Disparaged ER team/outpatient doctor to others for missed findings later discovered on the floor	36.60%	47.93%	52.08%	50.41%	11.32%	1.65%	.0043
Friend-ed a patient on facebook	77.36%	86.78%	21.13%	12.40%	1.51%	0.83%	.0311
Dated a patient	81.51%	90.08%	16.98%	9.09%	1.51%	0.83%	.0327
Cheated on exam because no time to study	76.60%	88.43%	22.26%	11.57%	1.13%	0.00%	.0062
Asked student to perform medical/surgical procedure on patient perceived beyond skill level	71.32%	80.99%	26.42%	17.36%	2.26%	1.65%	.0455
Reported patient information (labs, test results) as normal when uncertain of the true results	65.28%	76.03%	30.19%	19.83%	4.53%	4.13%	.0430
Written or called in a prescription for self	72.45%	83.47%	21.13%	13.22%	6.42%	3.31%	.0182

Table 7 illustrates that for 9 of the 11 behaviors, a greater percentage of DUH PGY1s reported having participated in that activity than VMC/ECU PGY1s; whereas there were two behaviors for which there were a greater percentage of VMC/ECU responders reporting they had participated in or observed when compared with DUH PGY1s.

Finally, of the 375 respondents, 73.5 percent of VMC/ECU participants and 75.6 percent of DUH participants indicated that they had received either positive or negative feedback on their own professionalism.

### Qualitative results

Themes were identified in the analysis of the two open-ended questions. Qualitative data were analyzed in aggregate as initial analysis showed no major differences.

Responders reported that the most important professionalism issues relevant to clinical practice include:

(1) respect for colleagues and patients

(2) relationships with pharmaceutical companies (conflict of interest)

(3) balancing home and work life

(4) admitting mistakes or simply stating “I don’t know”

Representative comments for each issue identified include – (1) “losing our calm when faced with confrontational patients and their family members”, “offering treatment equally to all patients regardless of insurance status”, “talking about patients in a joking manner”, (2) “accepting drug rep gifts”, “feeling pressured by pharmaceutical companies” (3) “time management, taking care of patients instead of being with my family”, “knowing when it’s okay to go home”, “taking stress home with us” (4) “acknowledging mistakes and learning from them”, “asking for help when needed”.

Responders reported that professionalism can best be assessed by peers, by patients, through formal observation of non-medical work, and by noting the timeliness and detail of paperwork submitted (e.g., charts, other types of deliverables).

## Discussion

This study sought to examine the perceptions and behaviors of physicians at an important transition time: entry into their first year of graduate medical education. Our findings inform our understanding of some of the professionalism issues faced by this generation of learners. The analysis offers implications for teaching and assessing professionalism in medical education.

As incoming PGY1s, the participants’ attitudes about the professionalism of behaviors certainly predated GME, and perhaps could be attributable to experiences encountered during their medical school, undergraduate settings or even earlier. Given what we know about the training environment of medical schools themselves, it seems highly likely that most had exposure to the behaviors included in the survey as part of both formal and hidden curricula.

A majority of responders rated all 46 specified behaviors as unprofessional. A majority also reported they had observed or participated in these behaviors. Thus, even for the more controversial professionalism issues, this group of learners suggested that behavior they perceive is unprofessional is also prevalent.

There were 15 behaviors in which greater than 10% of responders reported having participated. For these same 15 behaviors, 10-92 percent reported them as “unprofessional.” (See Figure 1) Identifying behaviors that a great percentage of responders reported having participated in provides programs and institutions a foundation for teaching and selecting professionalism guidelines. Institutions can make their behavioral expectations explicit with references to policies, lists of do-s and don’t-s, sample cases vignettes and role modeling by faculty and senior residents. It may be helpful for institutions to be more intentional about ensuring their learners and employees identify institutional “norms,” understand why these behaviors are considered unprofessional, and know consequences of violating policies.

For a behavior like “discussing patient information in a public space” virtually all, 92%, rated it as “unprofessional”. Yet a fourth of responders indicated they had participated in this activity. If deemed not professional, and residents already recognize this, how can they be encouraged to make better choices?

In addition to the steps described above, it may be necessary to apply “on the spot correction” in these types of situations with faculty providing feedback regarding the behavior immediately after it occurs. Such correction will need to be applied discreetly and with due regard to good feedback techniques. Data like these provide a blueprint for conversations to clarify expectations, problem solve impediments, and define consequences for unprofessional behavior. If a given trainee displays willful disregard for patient confidentiality after feedback is provided, the behavior will require a higher, more formal level of response by the program director or some other internal specific mechanism [28]. The authors are intrigued with why an individual would participate in an activity that s/he considers unprofessional. Perhaps this is related to contributing factors such as peer pressure, the hierarchical structure of training, or the reluctance of these residents to speak out overtly. Could this be an issue of faculty behavior modeling or the culture of an institution? As seen in the data, a large portion of responders reported having observed behaviors they deemed unprofessional. This may create additional challenges to teaching (or even raising concerns of) professionalism if faculty themselves are engaging in “unprofessional” behavior.

Furthermore, what is the consequence of the dissonance which likely occurs when the resident participates in a behavior their conscience indicates is unprofessional? These residents already perceive these behaviors as unprofessional, but participate in it anyway. Does this numb their sense of rightness and wrongness and lead to an ethical recalibration making it even more likely for them to blur the lines between their sense of what is professional and their actions? Does this contribute to burnout as residents perceive their actions are at odds with their innate sense of professionalism?

### Gender

Findings indicate interesting differences by respondent gender. In every situation presented, men were more likely than women to rate the behaviors as professional. These findings are consistent with similar studies that examine gender-based differences in perception of various types of issues: how men and women differ in communication styles, competitiveness and attitudes [29,30]. Perhaps because men viewed these situations as consistent with professional values, they reported they engaged in these behaviors more frequently than women. It may be interesting to speculate on whether men chose to participate in these situations because they viewed these activities as neutral, or whether they adjusted their judgment on the professionalism of the activities to justify their participation. It may also be that women are less likely to report their participation in an activity they believe is unprofessional or don’t self assess their participation in the same way. For the two items that reached statistical significance ("making disparaging comments about other members of the healthcare team" and "not alerting a supervisor that a mistake was made"), women’s choices seemed more congruent with competencies in teamwork, communication, and error disclosure. These gender differences support that men and women have different views and approaches to life decisions and behaviors.

### Institution

PGY1s’ perceptions of and reported engagement in behaviors varied significantly between two institutions located in the same state just 90 miles apart. These participants had not yet been exposed to residency faculty or immersed in the GME institutional culture. Interpreting the meaning of these findings leads us to speculate whether the responses by institution may suggest certain qualities about each cohort, or may speak to the types of medical school graduates attracted or attractive to the institutions. It may also reflect their previous medical school learning environment, and whether those schools placed relatively more or less emphasis on the teaching and assessment of professionalism.

For both of the behaviors related to interaction with representatives of the pharmaceutical industry (“attending a pharma-sponsored dinner” and “using drug samples”) there was a statistically significant difference between institutions. A greater percentage of DUH responders rated these behaviors as unprofessional. Participants received the survey at DUH during new trainee Orientation, at the same time related policy and educational material regarding pharma was distributed. While they were not yet immersed in the institutional culture, they were exposed to DUH’s stringent policy regarding relationships with pharmaceutical companies. VMC/ECU participants received the survey a few weeks into training, after a brief orientation that did not include the sharing of policy or related literature on the institution’s stance on relationships with pharmaceutical companies. This might account for the difference in responses by institution.

With the exception of the pharmaceutically-related items, VMC/ECU participants were more likely to rate behavior as unprofessional as compared with DUH participants. DUH PGY1s were more likely to have participated in the listed unprofessional behaviors, with the exception of the pharma-related activities. We are uncertain why such differences were identified in the cohorts of learners attracted to and selected by each institution. We are unaware of how these differences may impact performance and development of professional competence during training and beyond.

### Qualitative analysis

Much of the qualitative findings corroborated the quantitative results. We were gratified that the most common professionalism theme identified by responders, was “respect for patients.” Respect is a broad construct reaching far beyond simple courteous and honest discussions with patients. Similarly, Karnieli-Miller et. al. found that medical students understanding of respect goes beyond any one act, behavior or attitude [31]. More than half of participants were neutral or rated as “professional” the specific item “having a personal conversation in a patient corridor” and more than half admitted having participated in this activity. We believe an ill patient and/or their family members could easily overhear this type of personal conversation and interpret it as “disrespectful.” As we struggle to define professional behavior, it becomes obvious that the use of simple and general professionalism guidelines such as “respect for patients” may be insufficient to describe the specific behaviors we wish to encourage or extinguish.

“Relationships with pharmaceutical companies” is a controversial topic inherent in contemporary medicine. Responders noted this in both their qualitative and quantitative responses. Greater than 40% of responders rated attending a drug rep sponsored dinner or using a drug rep sample for a patient/family member as unprofessional. However, 57% and 61%/10% have participated in these activities respectively. The ACGME and other groups have provided guidelines, not accreditation standards, on these allowing for varying stances on the topic by institution issues [32]. With regard to teaching and evaluating professionalism, the issue of interaction with industry is best addressed as a function of how the institution creates its own policies, how the institution holds all of its team members accountable to them, how residents are informed of them, and how real or perceived conflicts in clinical care are recognized and managed.

“Balancing home and work life” was identified by responders as a professionalism issue and may be a product of the times. To a large extent, our entering PGY1s are generationally “millennials” who value work life balance, or what they would prefer to term, “integration”. ACGME and society’s attention to duty hours and a “positive learning environment” has fostered an awareness and expectation that residents and physicians should not train in exhausting or stressful environments [33,34]. In addition, the growing sub-specialty options and “lifestyle” careers in medicine as well as advancements in technology have provided an alternative, to some extent, to extended work hours. Knowing this is a professionalism issue for current residents may enhance institutional efforts to provide career counseling, practice tips on fatigue mitigation and didactic instruction or references on strategies to enhance resiliency and career-life balance.

We also found it interesting that PGY1s, would offer that “admitting a mistake” or saying “I don’t know” is an important professionalism issue today. Training entities and accrediting agencies monitor residency programs for perceived “malignant” learning environments. ACGME finds GME programs out of compliance when residents feel intimidated to raise questions or concerns, or when “service” outweighs “education.” for learners. Yet, the newest generation of physicians raise this as an issue, perhaps reflecting their hesitation to admit when a mistake has been made or when they are simply unsure. 5% of responders admitted “reporting patient information as normal when uncertain of true results” and 7% reported having “performed medical or surgical procedures on a patient beyond perceived level of skill.” 28% and 19% respectively reported having observed such behavior. It is unclear if this is the result of a broad cultural pressure (beyond medical education) to always be right and not admit insecurities. Or is this unique to medical school graduates, who have clearly been “right” a majority of their lives, at least academically, having exceled enough to be accepted into and graduate from medical school? This could also be attributed to a medical culture that does not foster admitting mistakes or asking for help.

### Significance to practice

While the responsibility is on the learner or practicing physician to identify their deficiencies, training institutions can create a culture that makes it acceptable to admit fault or insecurity in knowledge and or skill. Rich learning opportunities can result from learners acknowledging what they don’t know. Perhaps more importantly, provider errors can lead to opportunities for improvements to institution-wide processes and safer patient care. Lastly, medical school and residency training prepare physicians for lifelong learning. Ideally physicians must accept that admitting mistakes can help one become even more competent.

Once behaviors that raise concern about one’s professionalism are identified, the greater challenge is determining how to assess and address any issues or deficiencies. Medical educators report there will need to be a greater emphasis on qualitative assessment, reflection and direct observation with a shared understanding of what is competent or professional behavior [35-37]. The participants’ response to how professionalism can best be assessed (i.e., “by peers”) suggests learner self-awareness, which is consistent with the literature [38] and required by ACGME.

The challenge in identifying, teaching and assessing professionalism also requires faculty development focused on institution-specific expectations for professional behavior. Faculty must have a shared understanding of professionalism and consensus on criterion based standards. Adoption of professionalism milestones should help facilitate this shared understanding, [35] especially if the individual specialties identify some congruence of behaviors and development stages in this area. The central importance of role modeling by faculty in development of professionalism cannot be overstated [39].

### Limitations

Data from two entering cohorts of new residents at two GME sponsoring institutions may not be generalizable to all entering PGY1s and all resident training institutions. Although voluntary and anonymous, participants may have told us what they thought we wanted to hear, and not their true perspectives. This may especially be the case because the 46 behaviors were predominately unprofessional in nature. The survey selected only 46 items to reflect contemporary situations and, therefore, asking respondents about different vignettes may have resulted in vastly different responses [40].

An additional limitation revolves around the lack of statistical correction for the extensive testing of individual survey items. As mentioned in the method section, the overarching goal of this study was to uncover specific issues with perceptions and behaviors of professionalism and this analysis was entered with no preconceived notions. As a result, the analysis was very exploratory in nature and was conducted with knowledge that some associations that appear significant in this paper may be spurious.

Lastly, participants’ ratings of vignettes and self-report of their observation of or previous participation in the listed behaviors may not correlate with their future behavioral choices, especially given their new identities as freshly minted physicians. As they achieve greater independence and increased opportunities to serve as role models for more junior learners, their perceptions and actions may change. It’s also the case that it may be more telling to look at graduating resident responses to understand institutional culture. It is the intent of the authors to follow these two cohorts of learners and determine whether such changes occur during residency training.

## Conclusion

Teaching and assessing professionalism is a critical component of medical education, yet understanding how to do this is a challenge. The challenge stems from the difficulty in defining professionalism and the disconnect between standards and recognition, and behavior. Knowing that a given behavior may be perceived as unprofessional does not necessarily persuade a trainee not to participate in it. This is discouraging in some ways, yet also reinforces the notion that “it’s not what we say, but what we do” that really matters. The role modeling of appropriate professional behavior, either by faculty members or peers, may be a powerful influence on whether a given trainee decides to participate in behaviors they perceive as unprofessional.

Despite the fact that our participants were at the same stage of their training, they viewed common situations very differently. Differences by gender and institution suggest the need for tailored teaching and assessment of professionalism based on systematic analysis of these variables at the institutional level.

We are only beginning to understand the possible connections between professionalism and patient care outcomes. If such a connection exists, we feel that training institutions have an obligation to ensure competence in professionalism before graduating their residents. Our finding that a universal understanding of what constitutes professional behavior may not exist is an important first step for all parties concerned.

## Competing interests

We have read and understood the BMC Medical Education policy on declaration of interests and have no relevant interests to declare.

## Authors’ contributions

KA and AN developed the research idea and study plan. All authors were involved in the collection and interpretation of the data. RS completed the statistical analysis. All authors were involved in the drafting and revising of the manuscript. All authors read and approved final draft.

## Pre-publication history

The pre-publication history for this paper can be accessed here:

http://www.biomedcentral.com/1472-6920/14/60/prepub
